# Helper Innate Lymphoid Cells in Human Tumors: A Double-Edged Sword?

**DOI:** 10.3389/fimmu.2019.03140

**Published:** 2020-01-29

**Authors:** Nicola Tumino, Paola Vacca, Linda Quatrini, Enrico Munari, Francesca Moretta, Andrea Pelosi, Francesca Romana Mariotti, Lorenzo Moretta

**Affiliations:** ^1^Department of Immunology, IRCCS Bambino Gesù Children's Hospital, Rome, Italy; ^2^Department of Pathology, IRCCS Sacro Cuore Don Calabria, Negrar, Italy; ^3^Department of Laboratory Medicine, IRCCS Sacro Cuore Don Calabria Hospital, Negrar, Italy

**Keywords:** innate lymphoid cells, antitumor immune response, checkpoint inhibitors, natural killer cells, immunotherapy

## Abstract

Innate lymphoid cells (ILCs) were found to be developmentally related to natural killer (NK) cells. In humans, they are mostly located in “barrier” tissues where they contribute to innate defenses against different pathogens. ILCs are heterogeneous and characterized by a high degree of plasticity. ILC1s are Tbet^+^, produce interferon gamma and tumor necrosis factor alpha, but, unlike NK cells, are non-cytolytic and are Eomes independent. ILC2 (GATA-3+) secrete type-2 cytokines, while ILC3s secrete interleukin-22 and interleukin-17. The cytokine signatures of ILC subsets mirror those of corresponding helper T-cell subsets. The ILC role in defenses against pathogens is well-documented, while their involvement in tumor defenses is still controversial. Different ILCs have been detected in tumors. In general, the conflicting data reported in different tumors on the role of ILC may reflect the heterogeneity and/or differences in tumor microenvironment. The remarkable plasticity of ILCs suggests new therapeutic approaches to induce differentiation/switch toward ILC subsets more favorable in tumor control.

## Introduction

Innate lymphoid cells (ILCs) belong to a family of immune cells involved in innate host defenses against pathogens and tumors. In addition, they play an important role in lymphoid organogenesis and, after birth, in the remodeling and tissue repair of mucosal epithelial cells. Five major groups of ILCs have been defined on the basis of the pattern of cytokine production and developmental transcription factor (TF) requirements, namely, natural killer (NK) cells and the non-cytolytic “helper-ILC,” including ILC1s, ILC2s, ILC3s, and lymphoid tissue inducers (LTi) (1). Recently, Crome et al. described a new subset of ILC with regulatory function (regulatory ILC) (2). ILCs derive from a common CD34^+^ haemopoietic precursor expressing the ID2 TF (3, 4). Remarkably, the ratio between different ILC subsets generated from precursors varies depending on the source of CD34^+^ cells (e.g., umbilical cord blood vs. peripheral blood). In addition, the use of granulocyte-colony stimulating factor employed for CD34^+^ cell mobilization in hemopoietic stem cell transplantation adds a further degree of complexity in the generation of different ILC, a finding that should be taken into account for therapeutic use (5). In this context, it should be underlined that ILCs display a high degree of plasticity, not only during their differentiation but also in their mature compartments. Thus, a shift from one to another ILC subset has been well-documented and implies that their functional capability may change substantially in given microenvironments, particularly at tumor sites under the influence of soluble factors or cellular interactions (6). In addition to these polarizing signals in tissues, ILC plasticity depends on specific chromatin regions to be accessible to TFs, whereas regulatory regions controlling the expression of signature cytokine genes in CD4 T-helper cells become accessible only after activation by cytokine signals or infection. ILC regulatory elements are already poised or active before stimulation. This distinctive feature of ILCs suggests that ILC regulomes are highly prone to dynamic changes in response to the microenvironment (7).

ILCs are particularly enriched in barrier tissues such as gut, uterus, lung, and skin, suggesting that their main biological function is to respond rapidly to infections and to environmental or inflammatory signals (8–11). During the early phases of infections and/or tissue damage, ILC represent a suitable and rapid source of cytokines that play a critical role in the activation of immune responses, induction of neo-angiogenesis, and promotion of tissue repair and barrier integrity. Considering the particular set of cytokines produced, ILC subsets could play a role also in shaping the tumor microenvironment (TM) (12). Moreover, given their ability to produce large amounts of cytokines, ILCs may be important players in tumor-associated inflammation (13). While the role of NK cells in controlling tumor growth and metastasis is now well-established, the involvement of non-cytotoxic “helper” ILCs in tumors remains controversial and poorly understood. Studies in both murine experimental models and human patients are clearly needed to clarify whether ILCs may represent a reliable tool or a target for immunotherapy (14). NK cells are cytotoxic cells not only responsible for the innate cytolytic defenses against tumors and viruses, but they are also able to secrete proinflammatory cytokines, primarily interferon gamma (IFN-γ) and tumor necrosis factor alpha (TNF-α). The antitumor effect of NK cells is well-established and extensively reviewed in recent publications (15–17). The main NK-mediated mechanisms of defense are related to their cytolytic activity and to cytokine production. However, even their activity may be sharply inhibited in the TM due to inhibitory factors or PD-1/PD-L1 interactions (18–21). In addition, particular conditions, such as low NK/tumor cell ratios, may favor tumor escape (IFN-γ-induced HLA-I upregulation in tumor cells or induction of epithelial–mesenchymal transition) (22). Overall, however, NK cells play a positive role against tumors, primarily by controlling the tumor growth and metastatic spread.

Helper ILC classification into ILC1, ILC2, and ILC3 is made on the basis of their TF profile and the set of cytokine produced (1). Recently, the presence of helper ILC in different tumor types has been documented (13). However, the actual involvement of ILC in antitumor defenses or in tumor progression through their ability to regulate inflammatory processes, neo-angiogenesis, tumor proliferation, and formation of tertiary lymphoid organs is far from being elucidated.

## ILC1

ILC1s are Tbet^+^, secrete IFN-γ and TNF-α, and are distinguished from NK cells because they are not dependent on Eomes and lack cytolytic activity (23). Although the hallmark of ILC1 is their ability to produce IFN-γ, a CD49a^hi^ ILC1-like population, capable of lysing tumor cells, has been described by Dadi et al. in mouse mammary tumors expressing granzyme B and TNF-related apoptosis-inducing ligand and intermediate levels of Eomes (24). Analogously, another group identified two distinct subsets of ILC1s in murine tumors. These cells are CD49a^+^CD49b^−^Eomes^int^ or CD49a^+^CD49b^+^ Eomes^+^ ILC1-like cells, respectively. However, despite the presence of a lytic machinery, such ILC1s were found to be ineffective in controlling carcinogenesis and even potentially able to promote metastasis in a transforming growth factor beta (TGF-β)-rich TM. The authors suggested that these ILC1s could derive from NK cells and that TGF-β not only is responsible for such plasticity but can also suppress ILC1s antitumor function while promoting their proangiogenic effect (25).

In humans, CD56^+^CD16^−^ cells were found in solid tumors as well as in peritoneal and pleural fluids of cancer patients. Because these cells also expressed CD9 and CXCR3, they were thought to derive from NK cells acquiring an ILC1-like phenotype. They produced VEGF suggesting that, analogous to ILC1-like subsets present in mice, such NK-derived infiltrating ILC1s might favor tumor growth (26). An analogy between this ILC subset and decidual NK cells has also been proposed. Indeed, both in TM and in decidua the presence of factors, such as TGF-β, could induce the conversion of conventional NK cells into cells with proangiogenic and immune-tolerant features (27).

In Crohn's disease, interleukin (IL)-12 signaling has been shown to convert RORγt^+^ILC3s into RORγt^−^Tbet^high^ ILC1s and induce IFN-γ production. While the IL-12Rβ1 is expressed at comparable levels by all ILC subsets, ILC1s were found to be the only ILC subset expressing the IL-12 receptor B2 subunit. Thus, IL-12 may be a key driver for the production of effector cytokines by ILC1. In this context, it is noteworthy that intestinal barrier defects are usually associated with higher levels of IL-12 production by mononuclear cells of the *lamina propria* (23). The ILC1s production of proinflammatory cytokines such as IFN-γ and TNF-α supports the hypothesis that they mainly contribute to the progress and chronicity of inflammation thus favoring the malignant transformation (17). However, the role of ILC1s in the development of tumors or in the control of their growth remains ambiguous. Thus, it has been reported that IFN-γ, a key cytokine produced by ILC1, may display either a pro- or antitumorigenic effect. In particular, the antitumor effects of IFN-γ include its ability to recruit and activate effector immune cells (by the upregulation of costimulatory molecules, cytotoxicity, and cytokine production) and to inhibit tumor growth (induction of apoptosis). On the other hand, the protumorigenic effect of IFN-γ consists in the induction of tumor escape mechanisms through the upregulation of ligands for the major inhibitory checkpoints (i.e., PD-L1) and HLA class I molecules as well as induction of epithelial–mesenchymal transition. On the other hand, ILC1-derived cytokines may also be involved in antitumor immunity, suggesting that the ILC1 function may depend on the microenvironment context. In general, the effect of IFN-γ is related to the tumor type/microenvironment and to the intensity of IFN-γ signal (28, 29).

## ILC2

ILC2s express high levels of the TF GATA3 and are defined by their capacity to produce the type 2 cytokines IL-4, IL-5, and IL-13. ILC2s were shown to play a predominant detrimental role in various tumor settings (30). One of the first observations related to ILC2 and tumors was reported in 2014. In these studies, elevated ILC2 numbers and high levels of transcripts of ILC2-related genes including RORα, GATA3, and CRTH2 were found in peripheral blood of patients with gastric cancer (31). In addition, in acute promyelocytic leukemia, high numbers of ILC2 have been reported, which became activated upon sustained interaction of CRTH2 and NKp30 with their tumor-associated ligands (32). In acute promyelocytic leukemia, ILC2 could enhance the immunosuppressive activity of myeloid-derived suppressor cells (MDSCs) through IL-13 production (32). In line with these findings, an ILC2–MDSC immunoregulatory axis was described in human bladder cancer and in murine prostate tumors. In bladder cancer patients who underwent the standard intravescical *Bacillus* Calmette–Guerin (BCG) immunotherapy, high numbers of tumor-infiltrating ILC2 were detected and found to correlate with low T cell/MDSC ratios, and unfavorable prognosis (33). *In vitro*, BCG was shown to stimulate the production of IL-13 by ILC2, which, in turn, supported both the recruitment and the immunosuppressive activity of MDSC. This suggested that the ILC2/IL-13 axis could counteract the effectiveness of the therapeutic activity of BCG in this tumor (33). On the other hand, ILC2 might also be involved in antitumor immune responses. Indeed, Kim et al. reported that growth of lymphoma cells injected subcutaneously in mice could be inhibited by local overexpression of IL-33, a major cytokine promoting differentiation and function of ILC2s. In this model, it was shown that ILC2s mediate antitumor activity either by producing cytokines relevant in tumor control (IL-5, IL-13, and GM-CSF) or by producing CXCR2 ligands (CXCL1 and CXCL2), which could induce the apoptosis of CXCR2^+^ tumor cells (34). Although this study suggests an important antitumor role for ILC2s dependent on IL-33 activation, this role remains to be confirmed in a more physiological tumor setting, where IL-33 is not overexpressed.

A recent study investigated in murine models the role of ILC2s in both primary and metastatic lung tumors. It was reported that mice genetically lacking ILC2s displayed a significantly increased tumor growth rate and a higher frequency of circulating tumor cells resulting in increased metastatic spread to distal organs. In addition, the same authors demonstrated that *in vitro* ILC2 coculture with tumor cells, T cells, and peptide-pulsed dendritic cells correlated with increased MHCI expression on tumor cells and elevated level of Granzyme B expression, resulting in T-cell-mediated enhanced killing activity of tumor cells (35). Thus, although type 2 responses, in general, and ILC2s, in particular, have been associated to tissue remodeling and establishment of a tumor-promoting environment, these findings suggest that, at least in particular instances, they may play a favorable role against tumors.

## ILC3

Among helper ILCs, ILC3s are RORγt^+^ and secrete IL-22, IL-17, IL-8, and TNF-α. They are critical for maintaining mucosal tissue homeostasis, and their dysregulation has been associated to chronic intestinal inflammation and cancer. An association between IL-23-driven gut inflammation and tumorigenesis has been reported (36). Since IL-23 plays an important role in ILC3 development, it is not surprising that, as suggested by some studies (34, 35), the ILC3-derived IL-17 and IL-22 may contribute to the development of colorectal cancers. In this context, transgenic overexpression of IL-23 in wild-type mice was shown to be sufficient to induce adenoma formation in an ILC3-dependent manner, partly through IL-17 production (37). In addition, the production of IL-22 by NKp46^−^ILC3 was shown to play a role in tumor maintenance in a bacteria-induced colon cancer model (38). A similar subset of NKp46^−^IL-22-producing ILCs has been described to be able to regulate the activity and the expansion of T cells present in the TM (2). In this context, IL-17 and IL-22 produced by ILC3 may counteract tumor growth by favoring the recruitment of CD8 T cells, NK cells, and neutrophils. On the other hand, they may favor tumor proliferation/metastasis by inducing macrophage polarization toward M2 and Treg recruitment (38–40).

ILC3s are also involved in promoting tissue remodeling/repair and in maintaining tissues homeostasis. In particular, LTi-like cells are a subset of ILC3s that have been associated to improvements of antitumor immunity, by facilitating the infiltration of leukocytes in the tumor. In a skin metastasis mouse model, IL-12 has been shown to initiate local antitumor immunity by stimulating NKp46^+^ LTi cells, which, in turn, induced upregulation of adhesion molecules in the tumor vasculature resulting in increases in leukocyte invasion (41). In non-small cell lung cancer, NKp46^+^ILC3s with LTi-like properties were consistently found at the edge of intratumor tertiary lymphoid structures (that they contribute to organize through the production of TGF-β) and correlated with the density of such lymphoid aggregates within the tumor. Importantly, their presence was associated with a better clinical outcome. Also in non-small cell lung cancer, ILC3s induced the expression of adhesion molecules by newly formed endothelial cells in the tumor (9). Since the presence of tumor-associated ectopic lymphoid-like structures appears to correlate with a better prognosis in different tumors and considering that ILC3 may induce ectopic lymphoid-like structures formation and promote lymphoid organogenesis, the analysis of TI-ILC numbers and function may acquire an important prognostic value (42). Also in breast cancer, the presence of RORγt^+^ ILC3s able to facilitate tumor invasion into lymphatic system has been reported, thanks to their ability to modulate the production of soluble factors present in the TM (43).

Similarly to what occurs for Th17 cells, also the involvement of ILC3s in cancer immunosurveillance is pleiotropic. Conflicting available data could reflect, at least in part, the heterogeneity of ILC3 subsets, the tissue of origin, and the type of microenvironment of different tumors.

## Inhibitory Checkpoints

While NK cell activation and function is under the control of inhibitory receptors (checkpoints) such as the HLA-I-specific KIR and CD94/NKG2A (expressed constitutively) and PD-1, limited information exists on checkpoints possibly expressed by ILC3. In this context, PD-1, a well-known checkpoint controlling T-cell activation, has recently been shown to control also NK cell function (19, 21, 44). Checkpoint inhibitors, such as anti-PD-1 blocking antibodies, are currently used in the treatment of different advanced solid tumors. The unexpected positive clinical outcome has changed substantially the prognosis of tumors considered otherwise incurable (45). Notably, the selection of patients suitable for treatment is mostly based on percentages of PD-L1+ tumor cells in the tumor biopsy. However, the evaluations is made on heterogeneous tumor samples, such as surgical specimens or biopsies (in some instances inadequate in numbers), or using different anti PD-L1 clones, which may substantially vary in their reactivity, thus leading to possible discordances and incorrect clinical decisions (46, 47). The physiological role of PD-1 is important since it is involved in the induction and maintenance of peripheral immune tolerance. In this context, PD-1 expression at the feto-maternal interface during the early phases of normal human pregnancy seems to be crucial for a successful pregnancy. Peculiar NK cells and ILC3 are present in human decidua where they appear to play a relevant role in controlling the balance between inflammation and tolerance, as well as in inducing neoangiogenesis, tissue remodeling, and placentation (48). Indeed, during the first trimester, decidual ILC3 expresses high levels of PD-1. Specifically, high percentages of PD-1 were detected on both LTi-like cells and NCR^+^ILC3. In addition, these cells were found to express/coexpress TIM-3, another immune checkpoint. Importantly, the PD-1 expression undergoes progressive reduction during pregnancy reaching significantly lower levels at the third trimester. In particular, the negative correlation between proportions of PD-1^+^ILC3 and the stage of pregnancy suggests a role for PD-1^+^ ILC3 in the control of early stages of implantation. Of note, PD-1^+^ ILC3 displays a reduced capacity to release cytokines as compared to the PD-1^−^ ILC3 population. Moreover, mAb-mediated PD-1 cross-linking results in inhibition of the production of IL-22, IL-8, and TNF-α by PD-1^+^ ILC3 (20).

The immune checkpoint-mediated inhibitory mechanisms on T-cell (and, in part, NK cell) function has been studied primarily in tumors. Along this line, we have recently shown that also different ILC subsets are present in pleural effusions (PEs) from primary (mesothelioma) or metastatic tumors (adenocarcinoma). Thus, besides NK cells, helper ILC including ILC1, ILC2, and ILC3 were also detected in PEs in cancer. Upon specific stimulation, ILCs produce their typical cytokines suggesting that they could mediate an antitumor effect. Notably, both PE-NK cells and PE-ILC3 may express functional PD-1, while tumor cells may express PD-L1, suggesting a PD-1-mediated inhibitory effect on cells with potential antitumor activity (19).

Besides pregnancy and tumor settings, PD-1 expression was reported in mice on ILC-committed progenitors, capable of generating ILC1s, ILC2s, and ILC3s and a small number of circulating NK cells (49, 50). PD-1 expression is lost upon differentiation but expressed on effector tissue resident ILC2s upon IL-33 stimulation, resulting in reduction in their ability to release cytokines (49, 51). In addition to PD-1, it was reported that ILC2s express PD-L1 during murine pulmonary infection, and their interaction with PD1-expressing CD4^+^ T cells favors Th2 polarization (52). While the role of PD-1 expression on ILC2s seems to be relevant mainly in infections, these findings suggest that blocking PD-1/PD-L1 axis in the context of cancer could also affect type 2 responses. The possible favorable or unfavorable contribution of this ILC2-mediated response to therapy with checkpoint inhibitors should be further explored to improve the efficacy of cancer treatment.

Another inhibitory checkpoint expressed by ILCs is OX40L, which could interact with its natural receptor (OX40) on Th1 or Th2 cells. In particular, OX40L^+^-ILC2 induce an expansion of Th2 cells and Treg, suggesting a dual role of ILC2 in the induction or regulation of immune response (53). It is known that inflammatory stimuli (i.e., TNF-like ligand 1A) upregulate OX40L on ILC3 that, in turn, induce Th1-type inflammatory responses *in vivo*. It is possible to speculate that, in the absence of inflammatory stimuli or in TM, ILC3 could not induce T-cell responses against tumor cells (54).

## Conclusions

A number of experimental evidences have highlighted the relevant role of cells of the innate immunity in the positive or negative regulation of tumor growth and metastasis. Thus, polarization of different innate cell types may determine their effect on tumor control (6). While the first seminal evidences of the consequence of cell polarization at the tumor site were provided by Mantovani and colleagues for tumor-associated macrophages (55), other innate cell types present in the TM were subsequently shown to favor tumor escape from antitumor immune responses. Among ILCs, ILC2 may shape downstream adaptive immunity and favor “negative” type 2 responses. Notably, mechanisms similar to those exploited by tumors to subvert immune responses are known to play a physiological role in certain tissue environments. This is evident in decidua where NK cells, ILC3, macrophages, and other myeloid cells are polarized toward suppressive functional activities. The recent finding that PD-1 may be expressed also by NK cells provided an important information for the usefulness of therapy with mAbs disrupting the PD-1/PD-L1 axis in tumors that have lost (partially or completely) HLA-Cl-I molecules, thus becoming undetectable by cytolytic T cells. Indeed, in HLA-Cl-I-defective tumors, the function of PD-1^+^ NK cells can be unleashed, thus restoring their antitumor activity. As shown recently, a further potentiation of NK cell function can be achieved by blocking KIRs and/or NKG2A (56, 57) or using a monoclonal antibody (Ipilimumab) able to block the interaction between CTLA4 and its ligand B7 (58, 59). Of note, in the haploidentical hemopoietic stem cell transplantation setting, this blocking may render “alloreactive” all donor NK cells. In addition, the plasticity of ILC could be better exploited to favor the generation of cell subsets useful for their antitumor activity. This may be achieved *in vitro* not only by inducing (with appropriate cytokines) differentiation of CD34^+^ progenitors toward given ILC but also by acting on mature ILC or their precursors.

In conclusion, although the recent years, thanks to immunotherapy, witnessed unprecedented progresses toward the cure of solid tumors and high-risk leukemia, it is evident that further progresses are required, which may be based on better knowledge of the pathophysiology of different cell types, including ILCs and on new combined therapies, to minimize the effect of tumor escape mechanisms (60).

## Author Contributions

All authors discussed together the general outline of the article and contributed to the elaboration of the final version of the manuscript. NT, PV, LQ, and LM wrote the first draft that was subsequently reviewed by EM, FMo, AP, and FMa.

### Conflict of Interest

The authors declare that the research was conducted in the absence of any commercial or financial relationships that could be construed as a potential conflict of interest.
